# Construction of BiOCl/Clinoptilolite Composite Photocatalyst for Boosting Formaldehyde Removal

**DOI:** 10.3390/ma14216469

**Published:** 2021-10-28

**Authors:** Yonghao Di, Xiangwei Zhang, Xinlin Wang, Shuilin Zheng

**Affiliations:** School of Chemical and Environmental Engineering, China University of Mining and Technology (Beijing), Beijing 100083, China; 18910320633@163.com (Y.D.); zhangxiangwei0613@126.com (X.Z.); wangxinlin8426@163.com (X.W.)

**Keywords:** BiOCl, clinoptilolite, formaldehyde, photocatalysis

## Abstract

Binary composite was synthesized via coupling BiOCl with alkali leached natural clinoptilolite (40B0/CN), which showed retarded recombination of photo-generated carriers. The clinoptilolite was pretreated with alkali leaching, resulting in a larger pore size and high cation exchange capacity. The modified clinoptilolite was more feasible for the growth of BiOCl and to promote the adsorption ability for formaldehyde (HCHO). In addition, the cation exchange capacity was conducive to anchor Bi^3+^, further leading to the reduction of the particle size of BiOCl. The carrier effect of alkali leached natural clinoptilolite promoted the amorphous transformation of BiOCl at low temperature, which simultaneously produced more distortions and defects in the BiOCl lattice. The 40B0/CN composite exhibited the superior light absorption ability with a narrower band gap. The photocatalytic degradation rate for HCHO of 40B0/CN under solar light reached 87.7%, and the reaction rate constant was 0.0166 min^−1^, which was 1.6 times higher than that of BiOCl. This paper gave a deep insight into photocatalytic technology to efficiently degrade formaldehyde.

## 1. Introduction

Owning to the increasing attention to the living environment, indoor air quality (IAQ) has traditionally been regarded as an important factor that affects the health of humans [1,2,3]. In particular, formaldehyde, as one of the most concerned volatile organic compounds (VOCs), has emerged as being one of the main reasons causing cancer due to its widespread source and high toxicity [4,5,6]. To date, massive research efforts have been devoted to the development of adsorption [7], biodegradation [8], thermal catalysis [9], and photocatalysis [10] technologies to remove formaldehyde. Due to uncomplicated processes and an efficient treatment effect, adsorption technology has recently been widely studied. As a kind of natural zeolite and an efficient and environmental adsorption material, natural clinoptilolite (NC) has received widespread attention owing to its abundant resources, low price, and its massive mesoporous structure, which could promote the diffusion and mass transportation of formaldehyde and active species [11,12]. However, its formaldehyde adsorption capacity is limited. On the other hand, when external environmental conditions change, formaldehyde molecules are easily desorbed from the surface of NC, resulting in secondary pollution. Therefore, to make NC more feasible for practical application, it is very necessary to endow NC adsorbents with formaldehyde degradation function to ensure the continuous purification ability of materials to formaldehyde.

Among various degradation technologies for formaldehyde purification, photocatalysis has been proven to be effective in degrading or mineralizing formaldehyde. Thereinto, BiOCl is an inexpensive and promising photocatalyst with the advantages of a suitable band gap (3.40 eV), its non-toxicity, and being cost-effective and environmentally friendly [13,14]. Moreover, BiOCl displays the typical layered structure, which is conducive to the separation and migration of electron and hole pairs, and so it shows better photocatalytic activity. Massive reports have been proved that formaldehyde could be degraded by BiOCl under ultraviolet or solar light irradiation [15,16]. However, it is currently constrained by the shortcoming of lower degradation efficiency under solar light irradiation and tendency to form larger particle size, which inevitably decreases the number of active sites [17]. In view of the large specific surface area, NC is a promising carrier candidate for BiOCl to construct a coupling system with a higher formaldehyde adsorption capacity, adsorption selectivity, and efficient formaldehyde degradation performance. However, the smaller pore size of NC may hinder the diffusion of BiOCl into the holes. Previous studies have been proved that alkali leaching process could enlarge the pore size significantly [18,19]. Predictably, the NC after alkali leaching treatment would be more feasible for the deposition of BiOCl. Based on the above assumptions, combining alkali leached natural clinoptilolite and BiOCl is a satisfactory strategy to enhance the removal of formaldehyde, which would not only possess superior adsorption performance, but also exhibit formaldehyde degradation performance.

Overall, the BiOCl/alkali leached natural clinoptilolite was successfully prepared via facile liquid-phase hydrolysis method and well characterized by various instruments. The catalytic performance was examined by degrading formaldehyde in different systems. Meanwhile, the effect of preparation parameters such as reaction temperature and mass ratio of BiOCl were studied as well. Besides, the mechanism of the composite was fully explored and proposed.

## 2. Experimental

### 2.1. Materials

Sodium hydroxide (NaOH), bismuth nitrate (Bi(NO_3_)_3_), glycol (C_2_H_6_O_2_), sodium chloride (NaCl), and formaldehyde solution (37 wt%) were purchased from Beijing Reagent Co. (Beijing, China). All the above reagents were analytical reagent grade without any further purification. The natural clinoptilolite was provided by Bayannur City, Inner Mongolia Province, China. Deionized water was used throughout the experiments.

### 2.2. Composites Preparation

Alkali leached natural clinoptilolite (labeled as NC-Na-3.0) was prepared via impregnation method. In a typical synthesis, the NC and NaOH (3.0 mol/L) solutions were transformed into deionized water and the solid/liquid ratio was 1:10. The above suspension was further stirred for 6 h in a water bath at 60 °C. Then, the suspension was filtrated, dried in an oven (DHG-9240A, Huiyi Sifang Technical Service Co., Beijing, China) and collected for further use.

BiOCl/alkali leached natural clinoptilolite was synthesized through liquid-phase hydrolysis. Typically, 2.0 g NC-Na-3.0 was added into 61.42 mL Bi(NO_3_)_3_ ethylene glycol solution with the molar concentration of (m = 0.0125, 0.025, 0.0375, 0.05, 0.0625 mmol/L) under magnetic stirring to form a homogeneous suspension, then the suspension was continuously stirred in a water bath under different temperatures. After that, 92.13 mL sodium chloride solution with the same molar concentration of Bi(NO_3_)_3_ was added into the above suspension through the peristaltic pump (BT100M, Chuangrui Pump Co., Baoding, China), and finally the suspension was further reacted for 1 h. Then, the powder was collected after washing and drying. The composites with a different mass ratio of BiOCl were marked as XBY/CN (X and Y present the mass ratio of BiOCl and reaction temperature, respectively. X = 10%, 20%, 30%, 40%, and 50%, Y = 0, 25, 50, 75, and 90 °C). Moreover, pure BiOCl (labeled as B25 and B0) was obtained according to the abovementioned method at 25 °C and 0 °C water bath, respectively, without using NC-Na-3.0.

### 2.3. Characterizations

D8 advance X-ray diffractometer (Bruker, Karlsruhe, Germany) equipped with Cu-Kα radiation (λ = 0.154056 nm) was applied to investigate the phase structure of as-prepared composite. The range of 2θ was from 5° to 80° with a scanning rate at 8°/min. The morphology was studied on a scanning electron microscopy (SEM) (S-4800, Tokyo, Hitachi, Japan). The optical performance of composite was recorded through a UV-vis spectrophotometer (UV-9000s, Metash Instruments Co., Shanghai, China). The specific surface area and pore distributions were obtained by JW-BK (JWGB Sci. & Tech., Beijing, China) at liquid nitrogen temperature (77 K). Photoluminescence (PL) spectra was recorded through a fluorescence spectrophotometer (F-7000 PL, Tokyo, Hitachi, Japan) at 360 nm emission wavelength.

### 2.4. Evaluation of Photocatalytic Activity

One gram of as-prepared composite was evenly dispersed at the bottom of a glass-surface vessel (φ = 10 cm). Then, the glass-surface vessel was put on the support platform in the reaction chamber with a volume of 60 L. After that, the reaction chamber was closed and 20 μL formaldehyde diluent (18.5 wt%) was injected through a micro syringe. The electric heating plate and fan were employed to accelerate the volatilization of formaldehyde and finally the formaldehyde was evenly dispersed in the reaction chamber. After 2 min, the electric heating plate was turned off. The temperature was maintained at room temperature and the relative humidity maintained at around 50% (when testing under high humidity conditions, the relative humidity was maintained at about 75%). Before the simulated sunlight irradiation, 45 min of dark reaction was conducted to achieve the adsorption-desorption equilibrium on the surface of the composites. Then, the catalytic process was examined with a light intensity of 50 mW/cm^2^ at the sample surface. Generally, the degradation rate of formaldehyde was equal to the formation rate of CO_2_, thus the degradation rate of formaldehyde could be calculated according to the following formula:

(1) $$D=\frac{M_{1}\times △{\mathrm{CO}}_{2}}{M_{2}\times C_{0}}.$$

M_1_ and M_2_ represent the relative molecular weights of formaldehyde and carbon dioxide, respectively. △CO_2_ is the increment of CO_2_ (mg/m^3^). C_0_ represents the initial concentration of formaldehyde (mg/m^3^), and the theoretical calculation value is 66.8mg/m^3^. The concentration of CO_2_ and formaldehyde was monitored through a photoacoustic spectrum.

## 3. Results and Discussion

### 3.1. Cation Exchange Effect of Catalyst

Since NC possessed a superior cation exchange effect, it is necessary to further investigate whether the cation exchange performance is conducive to the growth of BiOCl, which is important for understanding the mechanism of the catalyst. A certain amount of NC-Na-3.0 was transferred into Bi(NO_3_)_3_ ethylene glycol solutions and the suspension was dispersed evenly under vigorous stirring. Then, the suspension was transferred to a water bath at 0 °C and 90 °C, respectively. After stirring for 2 h, the suspension was filtrated and dried, then the as-prepared composites were marked as CN-0 and CN-90, respectively. The chemical components of the above two samples were further analyzed. As shown in Table 1, the content of Bi in CN-0 and CN-90 was higher than NC-Na-3.0, while the content of Na and Ca in CN-0 and CN-90 are significantly lower than NC-Na-3.0, indicating that ion-exchangeable reaction between the Na^+^, Ca^2+^ in NC-Na-3.0 and Bi^3+^ was carried out. Moreover, there is little difference in the chemical components between CN-0 and CN-90, indicating that a low temperature could hardly inhibit the ion exchange between Na^+^, Ca^2+^ in NC-Na-3.0 and Bi^3+^.

### 3.2. Phase Structure of Catalysts

**Figure 1:**
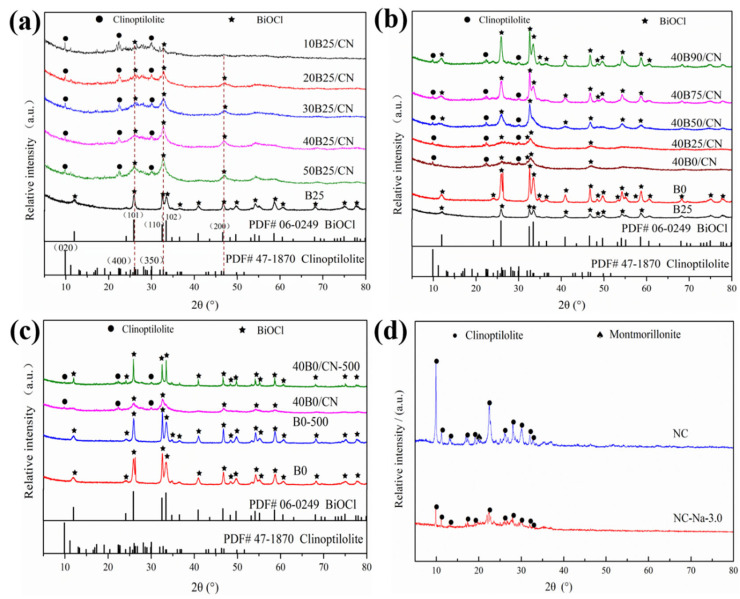
(**a**) XRD spectra of B25 and XBY/CN with different BiOCl loading amounts; (**b**) different reaction temperature; (**c**) XRD spectra of B0 and 40B0/CN before and after calcination; (**d**) XRD spectra of NC and NC-Na-3.0.

The phase structure of XBY/CN composites and the contrastive samples were investigated by XRD spectra. As shown in Figure 1a, the spectrum of B25 was basically consistent with the standard spectrum of BiOCl (JCPDS: 06-0249), indicating that the purity and crystallinity of BiOCl in B25 synthesized at room temperature were high [20]. The peaks appeared at 26.0°, 32.5°, and 46.8°, and could be indexed as (101), (110), and (200) planes of BiOCl in XB25/CN, respectively, and the peak intensity gradually enhanced with the increase of the loading ratio of BiOCl. Meanwhile, the peaks located at 26.0°, 32.5°, and 46.8°, which were attributed to (101), (102), and (200) planes of clinoptilolite, were gradually decreased. This implies that more BiOCl were successfully grafted on the surface of NC-Na-3.0. Moreover, the peak relative intensity of (110) crystal face of BiOCl in XBY/CN was higher than (101) crystal face of B25 and standard spectrum of BiOCl, which revealed that BiOCl presented an evident (110) preferred orientation on the surface of NC-Na-3.0.

The XRD spectra of 40BY/CN composites under different reaction temperatures were shown in Figure 1b. As can be seen, compared with B25, the diffraction peaks of BiOCl in B0 were significantly sharpened and the intensity was improved as well, indicating that the lower temperature was beneficial to increasing the crystallinity of BiOCl. However, the peaks’ intensity was enhanced gradually with the increased reaction temperature, indicating that the existence of NC-Na-3.0 exhibited a significant impact on the crystallization and actual loading amount of BiOCl.

Furthermore, the XRD spectra of B0 and 40B0/CN composites after calcination at 500 °C for 1 h (B0-500, 40B0/CN-500) was further studied to confirm the carrier effect of NC-Na-3.0. As shown in Figure 1c, the diffraction peaks of B0 did not change significantly after calcination, while the peak of BiOCl diffraction peaks in 40B0/CN were significantly sharpened and enhanced after calcination. This phenomenon could be attributed to the amorphous transformation of BiOCl at low temperature caused by the introduction of NC-Na-3.0. The induced transformation of NC-Na-3.0 might produce more distortions and defects in the BiOCl lattice, which would effectively promote the photocatalytic performance of the composites under solar light [21].

### 3.3. Pore Structure and Specific Surface Area of Catalysts

The specific surface area, pore volume, pore distributions of composites, and the average crystallite sizes of BiOCl in composites (calculated based on the Debye-Scherrer equation) are summarized in Table 2. NC-Na-3.0 possesses the largest specific surface area, total pore volume, and average pore diameter, which are beneficial for the adhesion and dispersion of BiOCl nanoparticles. Compared with B25 and B0, the specific surface area and pore volume of XBY/CN is improved, and the average pore size decreases apparently, suggesting that a large number of microporous pores were formed. When the mass ratio of BiOCl is small, the crystallite size of BiOCl loaded on NC-Na-3.0 carrier increases compared with B25, which may be due to the acid-base characteristics of the carrier surface. After alkali leaching treatment, more active centers that can react with acid remained on the mineral surface of natural clinoptilolite. These active centers would react with HNO_3_, which was produced by the hydrolysis of Bi(NO_3_)_3_ in NaCl aqueous solution, and finally the neutralization reaction could be realized. Therefore, the pH would be maintained to neutral, and the higher pH value promoted the rapid nucleation and growth of BiOCl. With the increase of the mass ratio of BiOCl, the crystallite size of BiOCl in XB25/CN gradually decreases as well as the total pore volume and average pore size (compared with NC-Na-3.0), indicating that the loading of BiOCl particles on the carrier may be completed preferentially in the pores of the carrier.

When the mass ratio of BiOCl was 40%, the smallest crystallite size of BiOCl and smaller average pore size of 40B0/CN appeared, which not only improved the photocatalytic activity of the composites but also facilitated the diffusion and mass transportation of reactants and active species. Besides, the uniformly BiOCl significantly improved the specific surface area and pore volume of 40B0/CN composite, and the surface area of 40B0/CN was 2.24 and 2.79 times as high as that of B0 and B25, respectively. It can also be seen from Table 2 that a higher reaction temperature is unfavorable to the formation of a smaller crystallite size of BiOCl.

### 3.4. Morphology and Structure of Catalysts

**Figure 2:**
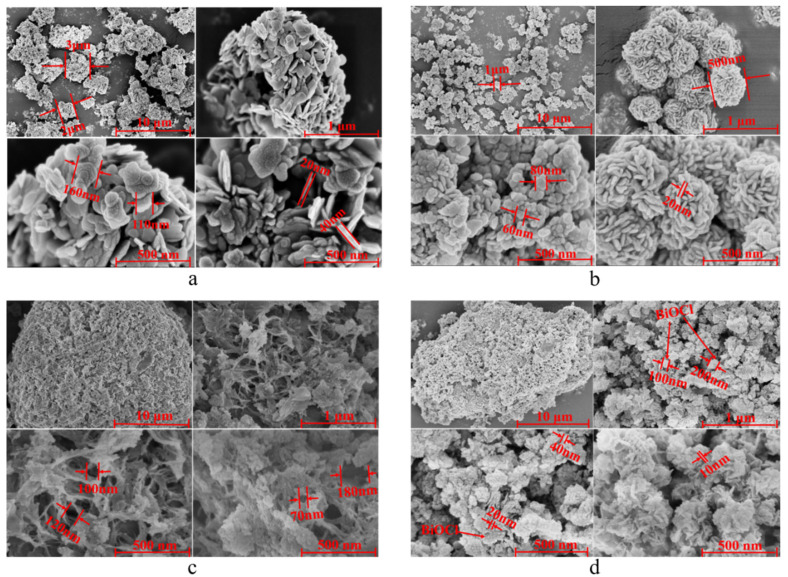
SEM images of pure BiOCl ((**a**) B25, (**b**) B0), clinoptilolite support ((**c**) NC-Na-3.0) and 40B0/CN (**d**).

The morphology and structure of as-prepared composites were investigated by SEM images. As shown in Figure 2, the B25 sample prepared at 25 ℃ exhibited a flake structure with a diameter of about 110~160 nm and a thickness of about 20~40 nm. The aggregate exhibit flake structure with a diameter of about 2~3 μm. The B0 sample prepared at 0 °C was mainly composed of flake structure with a diameter of about 60~80 nm and a thickness of about 20 nm. The BiOCl nanosheets were agglomerated into flower-like structure with a diameter of about 500 nm. As shown in Figure 2a,b, the particle size of B0 was significantly smaller than B25, and the size of B0 aggregates was small and uniform as well, which further indicated that a lower temperature could inhibit the growth of BiOCl and contribute to the dispersion on the surface of NC-Na-3.0. As shown in Figure 2c, the NC-Na-3.0 exhibited massive macropores with the pore size of 70~400 nm, while most of the original macropores of NC-Na-3.0 disappeared after loading of BiOCl (Figure 2d), which was ascribed to the growth of BiOCl in the macropores. As shown in Figure 2d, massive aggregates of BiOCl with the diameter of 100~200 nm and nanosheets of BiOCl with the diameter of 20~40 nm and a thickness of about 10 nm were dispersed on the surface of NC-Na-3.0. The smaller size of BiOCl might be caused by the carrier effect of NC-Na-3.0.

### 3.5. Optical Properties and Photoelectrochemical Performance of Catalysts

**Figure 3:**
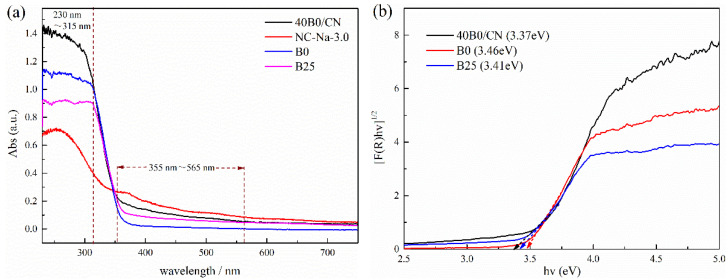
UV-Vis DRS (**a**) and band gaps (**b**) of different samples.

The optical performance of the as-prepared composites was measured via UV-Vis DRS spectra. The light absorption intensity of B25, B0, and 40B0/CN in the UV region, especially in the UV region of 230~315 nm, increased in turn, indicating that the smaller size of BiOCl was helpful to improve the utilization of ultraviolet light. In the region of >355 nm, especially in the region of 355~565 nm, the light absorption ability of B0, B25, and 40B0/CN increased in turn, indicating that the lower crystallinity of BiOCl might facilitate improving the utilization of visible light. In addition, the band gap values (Eg) were calculated using the Kubelka–Munk method [22], and the results were displayed in Figure 3b. The band gap of B25, B0 and 40B0/CN were estimated as 3.41 eV, 3.46 eV, and 3.37 eV, respectively. The narrower band gap of 40B0/CN further revealed that more visible light could be harvested.

### 3.6. Photocatalytic Activity and Stability of Photocatalysts

**Figure 4:**
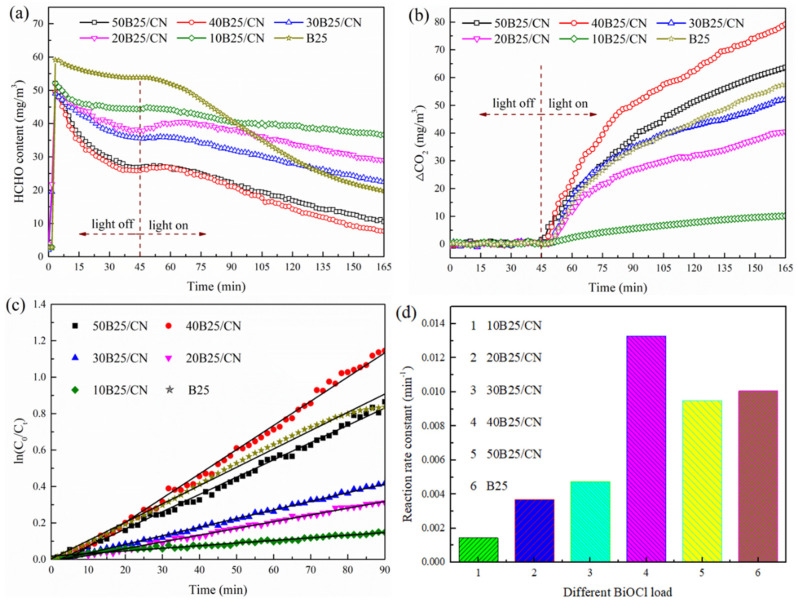
Variations of HCHO concentration (**a**), corresponding increased CO_2_ concentration (△CO_2_) (**b**), the kinetic curves (**c**) and the reaction rate constant values (**d**) over different samples under solar light.

Since the mass ratio of BiOCl could directly affect the catalytic performance of XB25/CN composites, the degradation rate of formaldehyde with a different loading amount of BiOCl was studied systemically. As depicted in Figure 4a, the concentration of HCHO showed a declining trend with the extension of time under dark condition, and gradually reached adsorption equilibrium within 45 min. Moreover, the concentration of CO_2_ remained almost unchanged during the dark adsorption process, which demonstrated that the decrease of HCHO was mainly attributed to the materials’ adsorption rather than degradation. B25 exhibited the smallest reduction of HCHO, resulting from poor adsorption ability. The XB25/CN composites possessed the higher adsorption of HCHO, which revealed that the introduction of NC-Na-3.0 significantly enhanced the adsorption performance. As shown in Figure 4b, the concentration of CO_2_ under different systems increased significantly, and the formation efficiency increased with the increase of BiOCl loading from 10% to 40%, and decreased when the BiOCl loading was excessive (X > 40%), suggesting that higher BiOCl loading amount was not conducive to the degradation of formaldehyde by composites. The corresponding first-order kinetics plot shown in Figure 4c,d demonstrated that the 40B25/CN composite shows superior formaldehyde purification performance under sunlight and the degradation efficiency was higher than of pure BiOCl. The K value of 40B25/CN (0.0133 min^−1^) was 1.32 times higher than that of B25 (0.0101 min^−1^) [23,24], further indicating that the introduction of NC-Na-3.0 significantly improves the photocatalytic performance of BiOCl.

**Figure 5:**
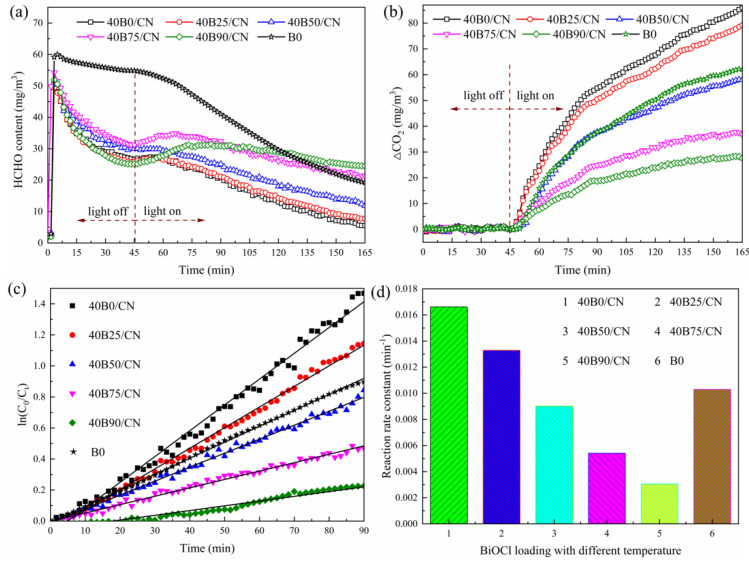
Variations of HCHO concentration (**a**), corresponding increased CO_2_ concentration (△CO_2_) (**b**), the kinetic curves (**c**) and the reaction rate constant values (**d**) over different samples under solar light.

It can be seen from Figure 5, that the formation efficiency of CO_2_ gradually increased with the decrease of reaction temperature. Moreover, the reaction rate constant gradually increased as well, which indicated that the lower reaction temperature was conducive to the improvement of the catalytic performance of B/CN. Specifically, when the reaction temperature decreased to 0 °C, the composite displayed the faster formation rate of CO_2_, and the K value of 40B0/CN (0.0166 min^−1^) was 1.61 times higher than that of B0 (0.0103 min^−1^). Thus, 40B0/CN was selected for further use in this work.

**Figure 6:**
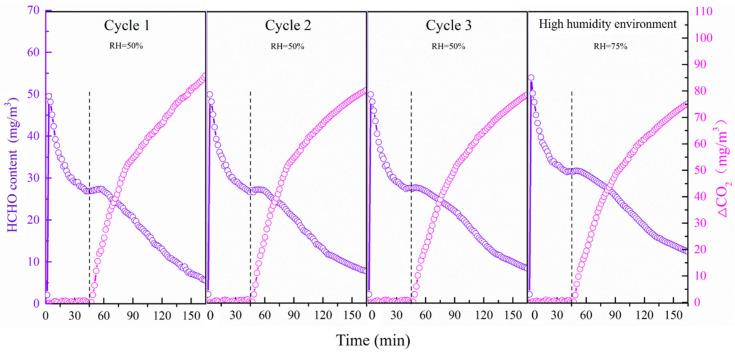
Repeated formaldehyde degradation performance of 40B0/CN in sunlight and its formaldehyde degradation performance in a high humidity environment.

The stability of photocatalytic performance is of great significance to the practical application of photocatalyst. The stability mentioned in this work includes the reusability of 40B0/CN and the photocatalytic performance of HCHO under high humidity conditions. It can be seen from Figure 6 that the degradation rate of HCHO and the formation rate of CO_2_ have no obvious reduction after three reuse experiments of 40B0/CN, indicating that the composite possessed the superior sustainable degradation performance for HCHO under solar light. Additionally, there was a minor reduction of HCHO and CO_2_ in the high humidity environment, which further indicated that 40B0/CN displayed good moisture resistance and had the application conditions in the high humidity environment.

**Figure 7:**
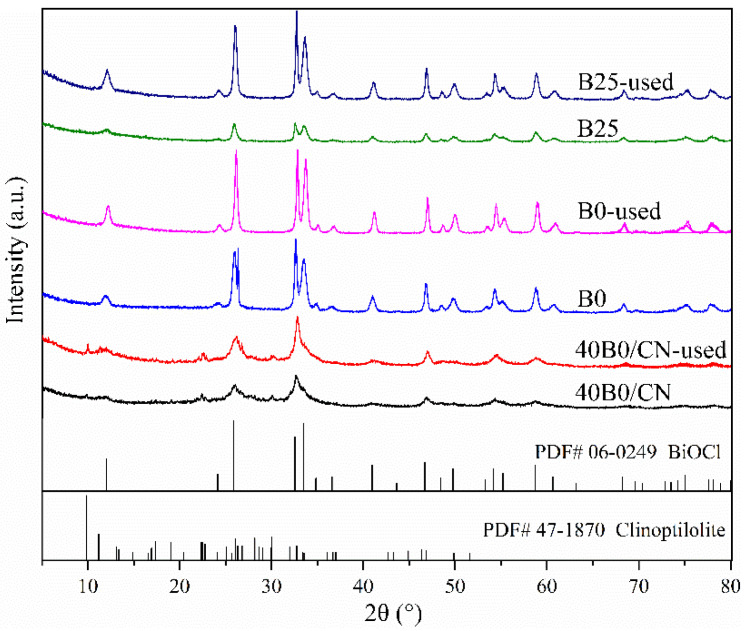
XRD spectra of B0, B25, and B0/CN before and after photocatalytic reaction under sunlight.

The XRD spectra of 40B0/CN, B0 and B25 before and after photocatalytic reaction were depicted in Figure 7. Obviously, the peak of BiOCl in B25 and B0 after the photocatalytic reaction was significantly sharpened and the intensity was enhanced, suggesting that the stability of the pure BiOCl was poor. The pure BiOCl synthesized by precipitation method had a smaller particle size. Moreover, the structural stability and crystallinity of it were generally lower than those of BiOCl after calcination. Therefore, it is also understandable that the structure and crystallinity changed after receiving light radiation energy. However, only slight changes were observed in 40B0/CN, which might be attributed to the existence of NC-Na-3.0, which could effectively maintain the stability of BiOCl.

### 3.7. Reaction Mechanism

**Figure 8:**
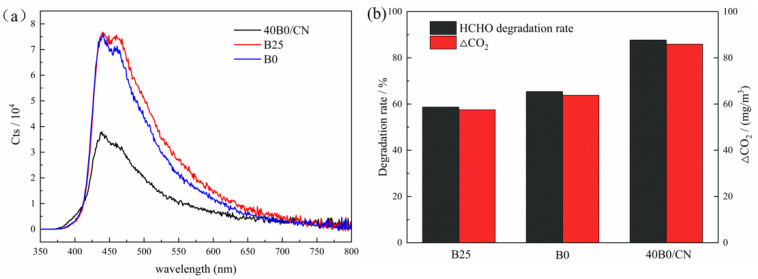
Photoluminescence spectra (**a**) and formaldehyde degradation rate under sunlight (**b**) of B25, B0 and 40B0/CN composites.

For the photocatalyst, the separation efficiency of photogenerated carriers was closely related to the photocatalytic activity. Based on the above analysis and discussion, it could be proved that the adsorption performance and photocatalytic performance of the composites were improved compared with BiOCl. The PL spectra was carried out to further investigate the separation efficiency of photogenerated carriers. As depicted in Figure 8a, the composites were excited under 290 nm and all of them revealed a wide emission peak from 350 to 800 nm. It is clearly observed that 40B0/CN showed weaker intensity than B25 and B0, which demonstrated that 40B0/CN possessed a lower recombination rate of electron-hole pairs [25,26]. This is consistent with the experimental results of the HCHO degradation rate in Figure 8b.

In conclusion, the preparation mechanism of 40B0/CN composite was as follows: After alkali leaching, NC-Na-3.0 obtained more large pores, and due to the existence of a strong electric field inside and around pores, more equilibrium ions Na^+^ and Ca^2+^ could be gathered. When NC-Na-3.0 was added into Bi(NO_3_)_3_ ethylene glycol solution, NC-Na-3.0 would carry out the ion exchange between Bi^3+^ and Na^+^, Ca^2+^ in the pores of NC-Na-3.0. Then, with the addition of NaCl aqueous solution, BiOCl preferentially nucleated and deposited around Bi^3+^ in the pores. Due to the confinement effect of the carrier, the growth of BiOCl would be inhibited, and the size of BiOCl would be reduced and the dispersion would be improved [27]. Moreover, the lower reaction temperature further decreased the deposition size of BiOCl on NC-Na-3.0 and further promoted its dispersion. The existence of NC-Na-3.0 also induced the transformation of BiOCl nanocrystals to an amorphous state.

The possible degradation mechanism was discussed, and the conclusion was depicted as three aspects: (1) Compared with pure BiOCl, the specific surface area of the 40B0/CN increased significantly and the adsorption of HCOH was significantly enhanced. The probability of collision between BiOCl particles and HCHO molecules could be greatly increased, which promoted the efficiency of photocatalytic degradation of HCHO; (2) More nano-scale BiOCl particles were evenly distributed on the surface of the carrier, and these particles have the tendency of amorphous transformation, resulting in increased distortion in the lattice. Thus, more visible light could be harvested by 40B0/CN, and the utilization of solar light was improved; (3) The combination of BiOCl and NC-Na-3.0 effectively promoted the separation and migration of photogenerated carriers, further improving photocatalytic performance.

## 4. Conclusions

The BiOCl/alkali leached natural clinoptilolite with superior photodegradation performance of gaseous formaldehyde were successfully prepared via facile liquid-phase hydrolysis method. There were a large number of BiOCl nanosheets with a diameter of 20~40 nm and a thickness of about 10 nm growth in the pores of NC-Na-3.0. The photocatalytic degradation rate of HCOH under solar light reached 87.7%, and the reaction rate constant was 0.0166 min^−1^, which was 1.6 times higher than that of BiOCl. Moreover, the composites possessed superior adsorption properties, good reusability, and moisture resistance. The introduction of NC-Na-3.0 as the carrier enhanced the adsorption performance for HCHO, promoted the directional deposition of BiOCl, and reduced the particle size of BiOCl. On the other hand, the improvement of dispersion and the induced transformation of BiOCl effectively reduced the band gap value, which enhanced the solar light absorption and boosted the separation and migration of photogenerated carriers.

## Figures and Tables

**Table 1:** X-ray fluorescence spectroscopy (XRF) results of clinoptilolite supports before and after ion exchange with bismuth nitrate at different temperatures (wt%).

Sample	SiO_2_	Al_2_O_3_	CaO	MgO	K_2_O	Na_2_O	Fe_2_O_3_	Bi_2_O_3_
NC-Na-3.0	61.39	21.46	5.35 ± 0.11	2.23	2.38	6.32 ± 0.13	0.83	0.01 ± 0.00
CN-0	64.31	22.37	3.39 ± 0.08	2.28	2.13	1.58 ± 0.06	0.63	2.86 ± 0.06
CN-90	64.25	21.92	3.23 ± 0.08	2.21	2.26	1.54 ± 0.06	0.85	2.76 ± 0.07

**Table 2:** Specific surface area, total pore volume, average pore diameter, and crystallite size of different samples.

Sample	Crystallite Size of BiOCl (nm)	S_BET_ (m^2^/g)	Total Pore Volume (cm^3^/g)	Average Pore Diameter (nm)
NC-Na-3.0	——	61.43	0.310	16.67
B25	19.5	29.29	0.160	15.63
10B25/CN	19.9	56.63	0.283	15.43
20B25/CN	12.5	45.29	0.181	13.61
30B25/CN	12.3	43.68	0.176	12.97
40B25/CN	12.2	42.55	0.162	12.38
50B25/CN	12.9	36.75	0.158	11.57
B0	18.6	23.89	0.148	15.84
40B0/CN	11.3	65.54	0.254	12.2
40B25/CN	12.2	42.55	0.162	12.38
40B50/CN	18.1	45.78	0.206	12.72
40B75/CN	23.3	50.82	0.209	13.52
40B90/CN	26.8	55.47	0.254	15.76

## Data Availability

The data presented in this study are available on request from the corresponding author.
